# Are preterm birth and very low birth weight rates altered in the early COVID (2020) SARS-CoV-2 era?

**DOI:** 10.3389/fped.2022.1093371

**Published:** 2023-01-09

**Authors:** Kayla Rodriguez, Matthew J. Nudelman, Priya Jegatheesan, Angela Huang, Kamakshi Devarajan, Jessica E. Haas, Rosemarie Cervantes, Kelle Falbo, Sudha Rani Narasimhan, Machelnil Cormier, Mary Beth Stewart, Rupalee Patel, Balaji Govindaswami

**Affiliations:** ^1^ Neonatology/Pediatrics, Mountain Health Network, Marshall University, Huntington, WV, United States; ^2^ Department of Pediatrics, Children’s Hospital at Montefiore, Bronx, NY, United States; ^3^ Neonatology/Pediatrics, Santa Clara Valley Healthcare, San Jose, CA, United States; ^4^ Neonatology/Pediatrics, St. Francis Medical Center, Lynwood, CA, United States; ^5^ Utilization Managment and Population Health, Silversummit Health Plan, Nevada Subsidiary of Centene Corporation, Las Vegas, Nevada, MO, United States; ^6^ VMC Foundation, San Jose, CA, United States

**Keywords:** SARS – CoV – 2, preterm, birth, very low birth weight, extremely low birth weight

## Abstract

**Objective:**

We evaluated the prevalence of preterm birth (PTB) and very low birth weight (VLBW) during Jan-Dec 2,020 (early COVID era) at 5 hospitals (2 in West Virginia, 3 in California) compared to Jan 2017–Dec 2019 (pre-COVID) inclusive of 2 regional perinatal centers (1 in Huntington, WV and 1 in San Jose, CA) and 3 community hospitals (1 each in Cabell, Los Angeles and Santa Clara counties).

**Design/methods:**

We examined PTB and VLBW rates of live births at 5 US hospitals from Jan 2017–Dec 2020. We compared PTB and VLBW rates in 2020 to 2017–2019 using Poisson regression and rate ratio with a 95% confidence interval. We stratified live births by gestational age (GA) (<37, 33–36, and <33 weeks) and birth weight (≤1,500 g, >1,001 g to ≤1,500 g, ≤1,000 g). We examined PTB rates at 4 of the hospitals during Jan-Dec 2020 and compared them to the prior period of Jan 2017–Dec 2019 using Statistical Process Control (SPC) for quarterly data.

**Results:**

We examined PTB and VLBW rates in 34,599 consecutive live births born Jan 2017–Dec 2019 to rates of 9,691 consecutive live births in 2020. There was no significant change in PTB (<37 weeks GA) rate, 10.6% in 2017–2019 vs. 11.0% in 2020 (*p* = 0.222). Additionally, there was no significant change when comparing VLBW rates in 2017–2019 to 2020, 1.4% in 2017–2019 vs. 1.5% in 2020 (*p* = 0.832).

**Conclusion:**

We found no significant change in the rates of PTB or VLBW when combining the live birth data of 5 US hospitals in 3 different counties.

## Introduction

Early in the SARS-CoV-2 pandemic, regional preterm birth (PTB) prevalence of very low birth weight (VLBW) infants in a designated Irish region reported temporal reduction from ∼8 to ∼2/1,000 live births (1). A similar reduction in extremely PTB from 2.19/1,000 to 0.19/1,000 during the nationwide lockdown was shown in a Danish study of live born singletons (2). Additionally, preterm birth rates of liveborn singletons born at 32–36 week GA were notably decreased following the lockdown implementation in China during Feb-May 2020 (3). In contrast, a pregnancy cohort of all births in two hospitals in Philadelphia did not show significant changes in singleton preterm or stillbirth rates (4). Stillbirths were no different in England either (5). Ten of the first twenty SARS-CoV-2 U.S. cases were reported in California (6) while West Virginia was the last US state to report the virus, several weeks after the first report on the West Coast. Our aim was to assess the PTB and VLBW rates of all live births at five US hospitals during the COVID-19 pandemic, including centers in California and West Virginia, to be representative of temporal variation in spread of the virus and all major racial, ethnic, and sociodemographic groups. Of note, all 3 US regions had undergone hospital bankruptcies, mergers and acquisitions during our study period.

## Methods

We examined PTB and VLBW rates of live births at five US hospitals, two in West Virginia and three in California, from Jan 2017 to Dec 2020. We compared PTB and VLBW rates in 2020 to 2017–2019 using Poisson regression and rate ratio with a 95% confidence interval. We stratified live births by gestational age (GA) (<37, 33–36, and <33 weeks) and birth weight (≤1,500 g, >1,001g to ≤1,500 g, ≤1,000 g). We examined PTB rates at four of the hospitals during Jan-Dec 2020 and compared them to prior period of Jan 2017–Dec 2019 using Statistical Process Control (SPC) for quarterly data.

## Results

**Figure 1:**
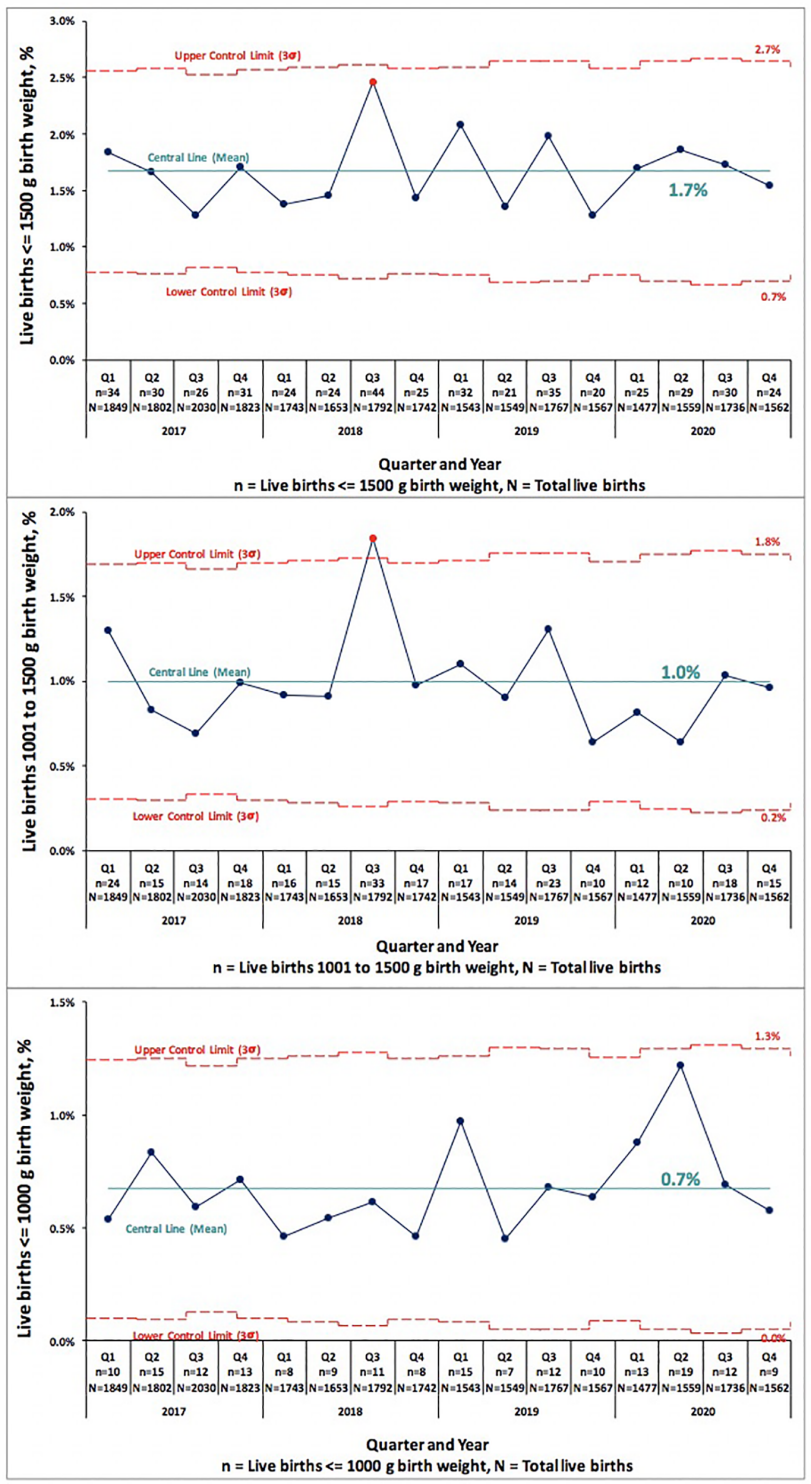
Pre-COVID-19 era (2017–2019) and COVID-19 era (2020) birth rates across five medical centers stratified by gestational age shown as statistical process control “p” chart. The central line represents mean and the upper and lower control limit lines are three standard deviations above and below mean.

**Figure 2:**
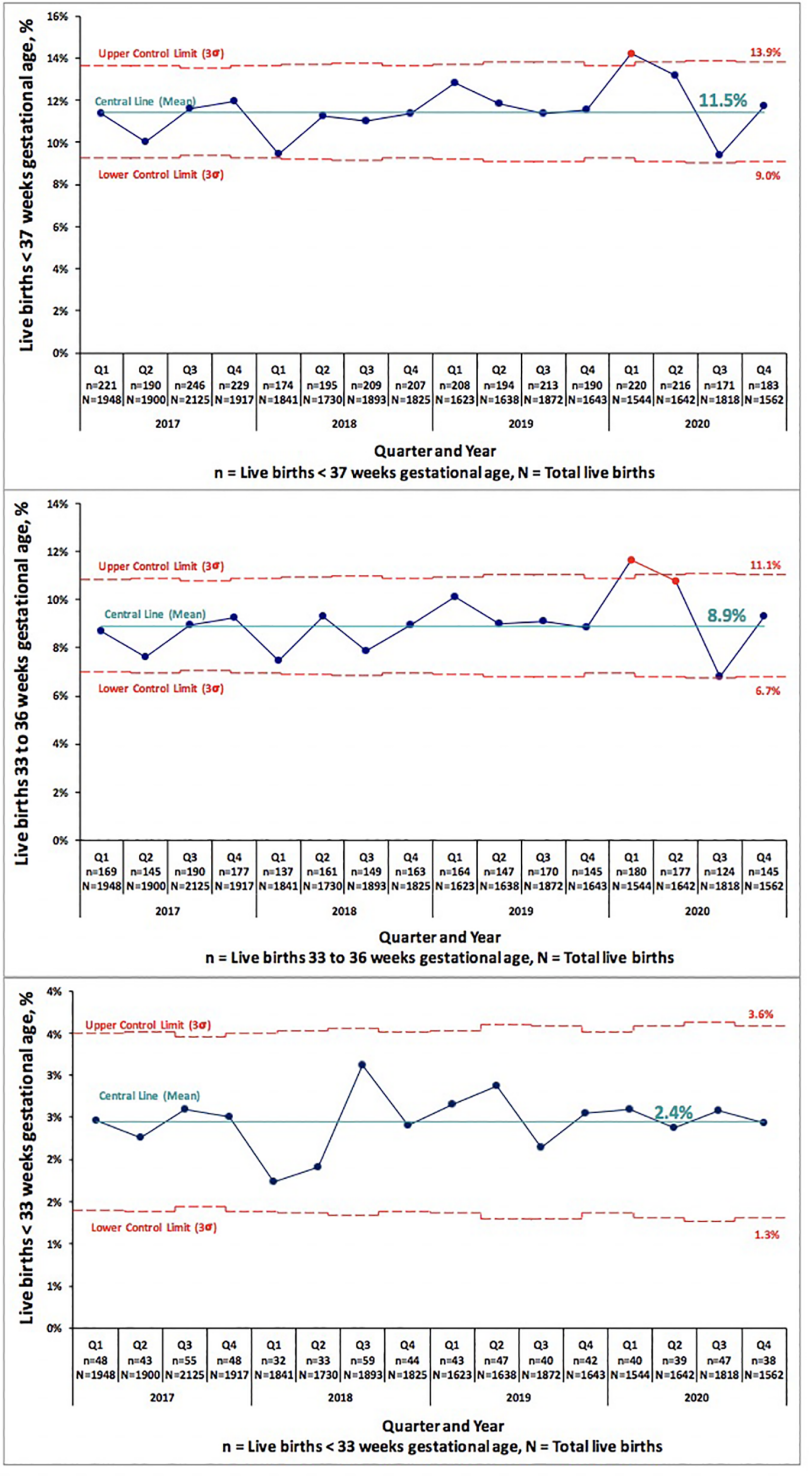
Pre-COVID-19 era (2017–2019) and COVID-19 era (2020) birth rates across five medical centers stratified by birth weight shown as statistical process control “p” chart. The central line represents mean and the upper and lower control limit lines are three standard deviations above and below mean.

We examined PTB and VLBW rates in 34,599 consecutive live births born Jan 2017–Dec 2019 to the rates of 9,691 consecutive live births in 2020. Table 1 compares PTB rates of 34,599 consecutive live births in 2017–2019 to 9,691 live births in 2020. There was no significant change in PTB (<37 weeks GA) rate, 10.6% in 2017–2019 vs. 11.0% in 2020 (*p* = 0.222). There was no difference in the subcategories of gestational age <33 or 33–36 weeks in any of the centers. Table 2 compares VLBW rates in 2017–2019 to 2020 and shows no significant change, 1.4% in 2017–2019 vs. 1.5% in 2020 (*p* = 0.832). There was no difference in the subcategories of birth weight 1,000–1,500 or ≤1,000 g in any of the centers except the regional NICU in San Jose, California showed an increase in ELBW (≤1,000 g) from 0.4% in 2017–2019 to 0.8% in 2020 (*p* = 0.013). Figures 1, 2 illustrate the quarterly birth rates in the birth weight and gestational age categories. The preterm birth rate in 2020 first quarter was an outlier due to an increase in the 33–36 weeks gestational age subcategory, although the subsequent rates are stable within the control limits. There are no outliers or significant shift in the overall birth rates in the gestational age or birth weight categories. The racial and ethnic distributions of all live births examined are as follows: 53% Hispanic, 30% Caucasian, 8% African American, 8% Asian, and 1% Other (data not presented).

**Table 1:** Comparison of pre-COVID era (2017–2019) vs. COVID era (2020) preterm birth rates across five medical centers stratified by gestational age (GA).

Center	GA, weeks	Year	*n*	Rate	Rate Ratio (95% CI)	*p* value
All centers^a^ (2017–19 *N* = 34,599; 2020 *N* = 9,691)	<37	2017–19	3,658	10.6% (10.2, 10.9)	0.96 (0.90, 1.03)	0.222
		2020	1,069	11.0% (10.4, 11.7)		
All centers (excluding St Francis Medical center)^b^ (2017–19 *N* = 21,955; 2020 *N* = 6,648)	<37	2017–19	2,476	11.3% (10.8, 11.7)	0.94 (0.87, 1.02)	0.160
		2020	794	11.9% (11.1, 12.8)		
	33–36	2017–19	1,917	8.7% (8.3, 9.1)	0.92 (0.84, 1.01)	0.075
		2020	630	9.5% (8.8, 10.2)		
	≤32	2017–19	534	2.4% (2.2, 2.6)	0.99 (0.83, 1.17)	0.874
		2020	164	2.5% (2.1, 2.9)		
Santa Clara Valley Medical Center San Jose, California (2017–19 *N* = 8,926; 2020 *N* = 2,780)	<37	2017–19	773	8.7% (8.1, 9.3)	0.91 (0.79, 1.05)	0.196
		2020	264	9.5% (8.4, 10.7)		
	33–36	2017–19	632	7.1% (6.5, 7.7)	0.94 (0.80, 1.10)	0.416
		2020	210	7.6% (6.6, 8.6)		
	≤32	2017–19	141	1.6% (1.3, 1.9)	0.81 (0.59, 1.11)	0.196
		2020	54	1.9% (1.5, 2.5)		
O'Connor Hospital San Jose, California (2017–19 *N* = 4,085; 2020 *N* = 950)	<37	2017–19	340	8.3% (7.5, 9.3)	0.98 (0.77, 1.24)	0.845
		2020	81	8.5% (6.9, 10.6)		
	33–36	2017–19	275	6.7% (6.0, 7.6)	0.93 (0.71, 1.21)	0.573
		2020	69	7.3% (5.7, 9.2)		
	≤32	2017–19	40	1.0% (0.7, 1.3)	0.78 (0.41, 1.48)	0.439
		2020	12	1.3% (0.7, 2.2)		
Cabell Huntington Hospital Huntington, West Virginia (2017–19 *N* = 7,849; 2020 *N* = 2,604)	<37	2017–19	1,327	16.9% (16.0, 17.8)	1.00 (0.90, 1.11)	0.992
		2020	440	16.9% (15.4, 18.6)		
	33–36	2017–19	974	12.4% (11.7, 13.2)	0.94 (0.84, 1.07)	0.367
		2020	342	13.1% (11.8, 14.6)		
	≤32	2017–19	353	4.5% (4.1, 5.0)	1.20 (0.96, 1.49)	0.119
		2020	98	3.8% (3.1, 4.6)		
St Mary's Medical Center^c^ Huntington, West Virginia (2017–19 *N* = 1,095; 2020 *N* = 314)	<37	2017–19	36	3.3% (2.4, 4.6)	1.15 (0.55, 2.38)	0.713
		2020	9	2.9% (1.5, 5.5)		
	33–36	2017–19	36	3.3% (2.4, 4.6)	1.15 (0.55, 2.38)	0.713
		2020	9	2.9% (1.5, 5.5)		
	≤32	2017–19	0			
		2020	0			
St Francis Medical Center^d^ Los Angeles, California (2017–19 *N* = 12,644; 2020 *N* = 3,043)	<37	2017–19	1,182	11.3% (10.8, 11.7)	0.94 (0.87, 1.02)	0.16
		2020	275	11.9% (11.1, 12.8)		

^a^ Combined data for all centers could only be described as GA <37 weeks because St Francis Medical Center did not provide stratified GA data.

^b^ St Francis Medical Center data did not provide stratified GA data thus was not included combined estimates.

^c^ St Mary's Medical Center does not care for infants <32 weeks GA, hence no data available for that GA group.

^d^ St Francis Medical Center provided GA data ranging between 22 and 36 weeks GA but there was insufficient data available to be able to stratify the GA.

**Table 2:** Comparison of pre-COVID era (2017–2019) vs. COVID era (2020) low birth weight rates across five medical centers stratified by birth weight.

Center	Birth weight, g	Year	*n*	Rate	Rate Ratio (95% CI)	*p* value
All centers (excluding St Mary's Medical center)^a^ (2017–19 *N* = 33,504; 2020 *N* = 9,377)	≤1,500	2017–19	476	1.4% (1.3, 1.6)	0.98 (0.81, 1.19)	0.832
		2020	136	1.5% (1.2, 1.7)		
	>1,000 and ≤1,500	2017–19	297	0.9% (0.8, 1.0)	1.15 (0.89, 1.49)	0.274
		2020	72	0.8% (0.6, 1.0)		
	≤1,000	2017–19	179	0.7% (0.5, 0.9)	0.78 (0.59, 1.04)	0.093
		2020	64	0.5% (0.5, 0.6)		
Santa Clara Valley Medical Center San Jose, California (2017–19 *N* = 8,926; 2020 *N* = 2,780)	≤1,500	2017–19	92	1.0% (0.8, 1.3)	0.73 (0.51, 1.07)	0.107
		2020	39	1.4% (1.0, 1.9)		
	>1,000 and ≤1,500	2017–19	56	0.6% (0.5, 0.8)	1.03 (0.60, 1.77)	0.926
		2020	17	0.6% (0.4, 1.0)		
	≤1,000	2017–19	36	0.4% (0.3, 0.6)	0.51 (0.30, 0.87)	0.013
		2020	22	0.8% (0.5, 1.2)		
O'Connor Hospital San Jose, California (2017–19 *N* = 4,085; 2020 *N* = 950)	≤1,500	2017–19	14	0.3% (0.2, 0.6)	1.09 (0.31, 3.78)	0.898
		2020	3	0.3% (0.1, 1.0)		
	>1,000 and ≤1,500	2017–19	10	0.2% (0.1, 0.5)	1.16 (0.25, 5.31)	0.846
		2020	2	0.2% (0.1, 0.8)		
	≤1,000	2017–19	4	0.1% (0.0, 0.3)	0.93 (0.10, 8.32)	0.948
		2020	1	0.1% (0.0, 0.7)		
Cabell Huntington Hospital Huntington, West Virginia (2017–19 *N* = 7,849; 2020 *N* = 2,604)	≤1,500	2017–19	240	3.1% (2.7, 3.5)	1.21 (0.92, 1.58)	0.177
		2020	66	2.5% (2.0, 3.2)		
	>1,000 and ≤1,500	2017–19	150	1.9% (1.6, 2.2)	1.38 (0.96, 1.99)	0.081
		2020	36	1.4% (1.0, 1.9)		
	≤1,000	2017–19	90	1.1% (0.9, 1.4)	1.00 (0.66, 1.50)	0.982
		2020	30	1.2% (0.8, 1.6)		
St Francis Medical Center Los Angeles, California (2017–19 *N* = 12,644; 2020 *N* = 3,043)	≤1,500	2017–19	130	1.0% (0.9, 1.2)	1.12 (0.74, 1.68)	0.594
		2020	28	0.9% (0.6, 1.3)		
	>1,000 and ≤1,500	2017–19	81	0.6% (0.3, 0.9)	1.15 (0.68, 1.93)	0.608
		2020	17	0.6% (0.3, 0.9)		
	≤1,000	2017–19	49	0.4% (0.3, 0.5)	1.07 (0.56, 2.06)	0.835
		2020	11	0.4% (0.2, 0.7)		

^a^ St Mary's Medical Center data did not provide birth weight-based data thus was not included combined estimates.

## Discussion

In the first year of the pandemic, 2020, we did not find a significant change in the rates of PTB or VLBW when combining the live birth data of five US hospitals in two different states and three different regions. No decrease in PTB or VLBW rates were noted at any of the five hospitals examined.

Similar results are noted in a study from France seeking to examine effect of lockdowns in perinatal outcomes in 2020, showing neither differences in preterm, nor stillbirth rates, nor LBW and adjusted VLBW rates in their cohort (7). In addition, preterm birth rates were reported unchanged in a large hospital system in Boston, MA in the USA during the first peak of the pandemic era (April-July 2020), secondary findings included no difference in spontaneous verse iatrogenic preterm birth rates (8). A more recent study from Australia showed decline in births <34 weeks GA during 2020–21 lockdowns but without significant change for births <28 weeks GA (9). A consortium of European nations, report decline in live birth rates, some with a subsequent rebound in early 2021 and excess COVID mortality as a putative reason for declining live births (10, 11). Recent (2020–21) Canadian surveillance data suggest increased preterm birth risk among 6,012 SARS-CoV-2–affected pregnancies (11.05% vs. 6.76%; relative risk, 1.63 [95% CI, 1.52–1.76]), inclusive of milder disease not requiring hospitalization, compared to unaffected contemporaneous pregnancies (12). A study from Bronx, New York, found preterm birth rates to be altered by the SARS-CoV-2 variant in women testing positive for SARS-CoV-2 during pregnancy (13). The rates of PTB were found to be lower during the Omicron variant surge when compared to the PTB rates during the original strain surge (13). These findings were attributed to differing variant virulence, increased vaccination availability, and improved SARS-CoV-2 management guidelines amongst other factors (13).

There was a significant increase in ELBWs in the San Jose regional hospital which was likely related to the change in referral patterns of high risk deliveries across the local county rather than the pandemic. The county acquired two community hospitals in early 2019 and the referral pattern for high-risk mothers were streamlined to deliver at the regional center. In addition, there was a local closure of the Labor and Delivery service at another community center that also redirected high risk mothers to the regional center.

As detailed above, multiple studies have been performed to analyze the effects of the COVID19 pandemic on perinatal outcomes and have shown conflicting results. A large rapid review and meta-analysis by Vaccaro et. al., was performed to examine the impact of the COVID-19 lockdown on the incidence of preterm birth, low birth weight, and stillbirth during the lockdown measures (14). When combining the data of 14 previous studies, the meta-analysis showed a significant risk of stillbirth during the COVID-19 lockdown when compared to the prepandemic period (14). However, PTB, LBW, and VLBW were not associated with a significant risk during the lockdown period (14).

Strengths of our study include a longer period of observation of all pregnancies into the early COVID era, a robust sample size with geographic, racial and ethnic heterogeneity. Limitations of our observations include lack of stillbirth data and inability to distinguish spontaneous vs. medically indicated preterm birth. Further limitations of our study include completing our study in 2020 thus lacking accurate SARS-CoV-2 infection rates and possibly largely excluding impact of vaccines and differing SARS-CoV-2 variants in pregnant women. In addition, local factors such as changes in referral patterns related to bankruptcies, mergers and acquisitions cannot be excluded.

## Conclusion

We conclude that in the first COVID-era year studied, 2020, SARS-CoV-2 did not reduce preterm or low birth weight rates in three different regions in the United States when combining the birth data of five US hospitals, compared to live births 2017–2019 at these same institutions. Local, regional and population-wide studies show variation in impact in SARS-CoV-2 on preterm birth rates, due to multitude of dynamic factors. These will have implications for planning and delivery of regionalized perinatal health care systems.

## Data Availability

The original contributions presented in the study are included in the article/further inquiries can be directed to the corresponding author.
